# Use of Practice-Based Research Network Data to Measure Neighborhood Smoking Prevalence

**DOI:** 10.5888/pcd10.120132

**Published:** 2013-05-23

**Authors:** Jeffrey A. Linder, Nancy A. Rigotti, Phyllis Brawarsky, Emily Z. Kontos, Elyse R. Park, Elissa V. Klinger, Lucas Marinacci, Wenjun Li, Jennifer S. Haas

**Affiliations:** Author Affiliations: Jeffrey A. Linder, Division of General Medicine and Primary Care, Brigham and Women’s Hospital, and Harvard Medical School, Boston, Massachusetts; Phyllis Brawarsky, Division of General Medicine and Primary Care, Brigham and Women’s Hospital, Boston, Massachusetts; Nancy A. Rigotti, Tobacco Research and Treatment Center and General Medicine Division, Massachusetts General Hospital, and Harvard Medical School, Boston, Massachusetts; Elyse R. Park, Tobacco Research and Treatment Center and General Medicine Division, Massachusetts General Hospital, and Harvard Medical School, Boston, Massachusetts; Emily Z. Kontos, Department of Society, Human Development and Health, Harvard School of Public Health, Boston, Massachusetts; Wenjun Li, Division of Preventive and Behavioral Medicine, University of Massachusetts Medical School, Boston, Massachusetts.

## Abstract

**Introduction:**

Practice-Based Research Networks (PBRNs) and health systems may provide timely, reliable data to guide the development and distribution of public health resources to promote healthy behaviors, such as quitting smoking. The objective of this study was to determine if PBRN data could be used to make neighborhood-level estimates of smoking prevalence.

**Methods:**

We estimated the smoking prevalence in 32 greater Boston neighborhoods (population = 877,943 adults) by using the electronic health record data of adults who in 2009 visited one of 26 Partners Primary Care PBRN practices (n = 77,529). We compared PBRN-derived estimates to population-based estimates derived from 1999–2009 Behavioral Risk Factor Surveillance System (BRFSS) data (n = 20,475).

**Results:**

The PBRN estimates of neighborhood smoking status ranged from 5% to 22% and averaged 11%. The 2009 neighborhood-level smoking prevalence estimates derived from the BRFSS ranged from 5% to 26% and averaged 13%. The difference in smoking prevalence between the PBRN and the BRFSS averaged −2 percentage points (standard deviation, 3 percentage points).

**Conclusion:**

Health behavior data collected during routine clinical care by PBRNs and health systems could supplement or be an alternative to using traditional sources of public health data.

## Introduction

Population distribution measurements of health indicators for chronic diseases can be used to target health care and community resources to areas with greatest need. The Centers for Disease Control and Prevention (CDC) and state health departments expend time, money, and energy measuring the distribution of health conditions and behavioral risk factors throughout the United States. The Behavioral Risk Factor Surveillance System (BRFSS), a cornerstone of these efforts with an annual budget of about $15 million, collects data on smoking, weight, diet, exercise, preventive medical care, and other behaviors (1,2). However, the BRFSS was designed to provide stable estimates at the metropolitan and state levels; because of limited sample sizes, creating stable estimates at the neighborhood level requires complex modeling and pooling several years of data (3).

Practice-based research networks (PBRNs) and integrated health delivery systems also collect patient behavioral health data in the course of routine clinical care (4,5). With increasing use of electronic health records (EHRs), behavioral data are more commonly available in standard formats (6). Data from larger health systems and PBRNs could provide a complementary or alternative method of monitoring neighborhood-level prevalence of behavioral risk factors.

Tobacco use remains the leading cause of preventable death in the United States (7). Knowledge about neighborhood-level smoking prevalence is necessary to guide the efficient deployment of community-based resources. Patient smoking status is routinely recorded in EHRs; EHR data derived from PBRNs and health systems may provide accurate community-level estimates of the prevalence of tobacco use. To determine if PBRN data could be used to make reasonable neighborhood-level estimates of smoking prevalence, we compared PBRN data to population-based, neighborhood-level estimates of smoking prevalence derived from the BRFSS.

## Methods

### Overview

Our analysis focused on 32 neighborhoods in the greater Boston area that corresponded to the catchment area of the Partners Primary Care PBRN. We compared population-based smoking prevalence estimates derived from the BRFSS and the US Census with 2009 prevalence estimates derived from the Partners Primary Care PBRN. We limited our analysis to respondents and patients aged 18 years or older. The Partners Human Research Committee approved the study protocol.

### Data extraction and data analysis

#### Three population-based smoking prevalence estimates

We derived individual-level data for population-based smoking prevalence estimates from the BRFSS, a population-based telephone survey administered by the CDC and state health departments (1). The BRFSS was designed for statewide or metropolitan area use and has been a primary source of population-based information on the prevalence of smoking. As part of the random-digit–dial survey, the BRFSS asks respondents, “Have you smoked 100 cigarettes in your lifetime?” and, if yes, “Do you currently smoke cigarettes?” We included annual population-based data from 1999 through 2009 for respondents who were at least 18 years old and living in 1 of the 32 neighborhoods in the Partners Primary Care PBRN catchment area, based on census tracts. The response rate for the BRFSS in Massachusetts in 2009 was 48%. Community-level data were derived from the 2000 Census.

We combined individual-level data from the BRFSS and community-level data from the US Census to estimate community-level smoking prevalence using a mixed-effects logistic regression model described previously (3). To summarize, using data from 1999 to 2009, the model predicted the prevalence of smoking in each of the 32 five-digit zip code levels in 2009. The model included 7 individual-level characteristics (age, sex, race/ethnicity, marital status, education level, employment, and annual household income), 8 community-level characteristics (median per capita income, percentage of owner-occupied housing units, percentage of blue-collar jobs in the total employed labor force, racial diversity, percentage of vacant housing units, percentage of population in rural area, crude rate of admission to Department of Public Health–funded substance abuse treatment programs, and density of tobacco outlets [number measured per mile of road]), and the year of interview.

#### Three PBRN-based smoking prevalence estimates

The Partners Primary Care PBRN includes 23 practices in eastern Massachusetts affiliated with Brigham and Women’s Hospital or Massachusetts General Hospital. The 23 practices include 5 hospital-based practices, 12 community-based practices, and 6 community health centers. We included patients aged at least 18 who made at least 1 visit to 1 of these PBRN practices in 2009 and had a zip code in 1 of the 32 neighborhoods in the catchment area of the PBRN. The Partners Primary Care PBRN practices use the Longitudinal Medical Record, an internally developed, web-based, fully functional EHR (8). We assessed smoking status using the EHR patient problem list and the health monitoring module, which contains concepts about health behaviors, prevention, and chronic disease monitoring. Information is entered into the problem list and health monitoring module by various members of the health care team.

We calculated the practice-based smoking prevalence and the frequency with which smoking status was not documented. We calculated the “market share” of the Partners Primary Care PBRN in each of the 32 neighborhoods by dividing the number of PBRN patients by the US Census population 18 years or older in that neighborhood.

We estimated neighborhood smoking prevalence in 2 ways using PBRN data. First, we calculated the crude prevalence of smoking in the PBRN data in each neighborhood by dividing the number of smokers by the total number of patients — smokers, nonsmokers, and patients with undocumented smoking status — from that neighborhood. Second, we calculated the smoking prevalence standardized by the neighborhood makeup according to the 2000 US census using age in 4 categories (18–39, 40–59, 60–79, or ≥80), sex, and race/ethnicity in 4 categories (white, Latino, black, or other). There were no patients in 71 of 1,024 cells. To make standardized estimates for these cells, we calculated predicted probabilities using a logistic regression model with smoking as the outcome and 2-way and 3-way interactions between age, sex, and race/ethnicity. We then filled the empty cells by calculating a smoothed estimate of the smoking prevalence by fitting the same logistic regression model but leaving out the 3-way interaction term for that cell.

### Statistical analysis

For BRFSS data, we calculated 95% confidence intervals using the variances of the random effects from the logistic regression model. For the PBRN data, we calculated exact binomial 95% confidence intervals. Because of the large sample sizes and our interest in how similar the prevalence of smoking was, we relied on clinical significance rather than formal statistical testing. For ease of interpretation and because smaller increments are unlikely to be clinically significant and may give a false sense of precision, we rounded all proportions to the nearest whole percentage. We assessed the relationship between the practice at which patients were seen and the neighborhood in which they lived using Cramer’s *V*. We considered a Cramer’s *V*, which can range from 0 to 1, of 0.25 or higher a strong relationship (9). We used ArcMap 10.0 (ESRI, Redlands, California) to generate maps, Stata 11.0 (StataCorp, LP, College Station, Texas) to obtain BRFSS population-based estimates, and SAS 9.2 (SAS Institute Inc, Cary, North Carolina) to calculate PBRN-based estimates.

## Results

### Sample sizes and characteristics

According to the US Census, 877,943 adults lived in the 32 neighborhoods in 2009 (population range across neighborhoods, 4,143–60,028). From 1999 to 2009, there were 20,475 adult BRFSS respondents who lived in the target area of interest used to estimate 2009 smoking prevalence (sample range across neighborhoods, 85–1,731).

There were 77,516 adult patients seen in the Partners Primary Care PBRN in 2009 who lived in 1 of the 32 neighborhoods (range across neighborhoods, 430–7,960). There was a strong relationship between the practice patients attended and neighborhood in which they lived (Cramer’s *V*, 0.33). Compared with census-determined characteristics, adults seen in Partners PBRN practices were more likely to be older, female, and Latino (Table 1).

**Table 1:** Patient Characteristics Derived From Population-Based Data and Practice-Based Research Network Data, 32 Neighborhoods in Boston, Massachusetts

Characteristic	2000 Census	Practice-Based Research Network

	N (%)	
**Total**	877,943 (100)	77,516 (100)
**Age, y**		
18–39	457,634 (52)	27,629 (36)
40–59	253,104 (29)	28,705 (37)
60–79	128,800 (15)	17,074 (22)
≥80	38,405 (4)	4,108 (5)
**Sex**		
Female	463,807 (53)	47,831(62)
Male	414,136 (47)	29,685 (38)
**Race/ethnicity**		
White	570,879 (65)	44,877 (58)
Black	114,940 (13)	8,449 (11)
Latino	83,701 (10)	15,272 (20)
Other	108,423 (12)	8,918 (12)
**Language**		
English	712,249 (69)^a^	63,463 (82)
Spanish	108,275 (10)^a^	9,401 (12)
Other	212,334 (21)^a^	4,652 (6)

a Language was available for the census only for the population age 5 or older. Total N for census language data was 1,032,858.

### Practice-based smoking documentation

Overall, 12% of patients seen at the Partners PBRN practices were documented smokers and 27% did not have smoking status documented. Across the 23 practices, the documented smoking rate averaged 11% (range, 4%–24%). The proportion of patients without smoking status documented across the 23 practices averaged 27% (range, 1%–79%).

### Neighborhood smoking prevalence

**Figure 1:**
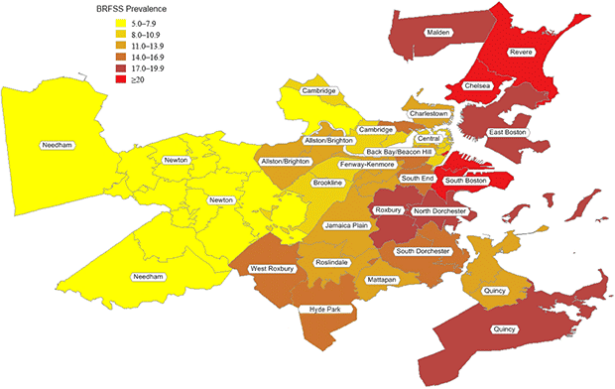
Population-based smoking prevalence in 32 Boston neighborhoods. Data derived from the Behavioral Risk Factor Surveillance System, 1999–2009. **Table:** 

According to the BRFSS, smoking prevalence averaged 13%, ranging from 5% to 26% (Table 2, Figure 1). Partners PBRN practices had a market share among adults in the 32 communities that averaged 10%, ranging from 3% to 32% (Table 2). Standardizing data according to the demographic makeup of a community resulted in modest changes in the estimated smoking prevalence: rates were higher in 16 neighborhoods, lower in 5 neighborhoods, and the same in 11 neighborhoods. The overall prevalence averaged among neighborhoods remained 11% (range, 5%–22%).

**Table 2:** Smoking Prevalence Estimated From the Behavioral Risk Factor Surveillance System and the Partners Primary Care Practice-Based Research Network, 32 Neighborhoods in Boston, Massachusetts

Community Name^a^	Adult Population^b^	Population-Based Estimates^c^		Practice-Based Research Network				Percentage Point Difference in Standardized Population Estimates
	
		N (1999 to 2009)	Smoking Prevalence, % (95% CI)	N (2009)	Market Share,^d^ %	Crude Smoking Prevalence,^e^% (95% CI)	Standardized Smoking Prevalence,^f^ % (95% CI)	
South Boston	24,569	872	21 (19–24)	1,723	7	12 (10–13)	11 (9–13)	−10
Cambridge (141)	10,204	85	15 (13–18)	495	5	8 (6–10)	8 (5–11)	−7
Quincy (169)	42,855	536	19 (16–21)	1,190	3	12 (10–14)	12 (10–15)	−6
North Dorchester	24,343	686	18 (16–21)	1,257	5	13 (11–15)	12 (10–14)	−6
South End	37,451	1,310	15 (14–17)	3,009	8	10 (9–11)	10 (9–11)	−5
Revere	37,363	426	26 (23–29)	7,960	21	20 (19–21)	22 (21–23)	−4
Cambridge (140)	13,907	218	9 (8–11)	638	5	5 (3–7)	6 (4–8)	−4
Back Bay/Beacon Hill	4143	394	12 (10–14)	714	17	8 (6–10)	9 (7–11)	−3
Chelsea	25,512	276	21 (18–24)	6,113	24	15 (14–15)	18 (16–20)	−3
Quincy (170/171)	29,789	336	14 (12–16)	970	3	10 (9–12)	11 (8–13)	−3
Needham	29,589	389	8 (6–9)	2,393	8	5 (4–6)	5 (4–6)	−2
West Roxbury	19,741	911	15 (13–16)	3,126	16	11 (10–12)	12 (10–14)	−2
South Dorchester	53,044	1,731	16 (14–17)	2,768	5	13 (12–14)	13 (12–15)	−2
Brookline	46,737	799	8 (7–10)	3,949	8	6 (6–7)	7 (6–8)	−2
Cambridge (139/142)	31,839	406	11 (9–12)	1,457	5	8 (7–10)	9 (7–11)	−2
Fenway-Kenmore	45,724	776	12 (11–14)	1,763	4	11 (10–13)	11 (9–13)	−1
East Boston	29,364	935	17 (16–19)	2,182	7	17 (15–19)	16 (13–20)	−1
Cambridge (138)	31,989	401	7 (6–9)	1,124	4	6 (5–7)	6 (5–8)	−1
Roxbury	32,908	1,060	17 (16–19)	3,100	9	16 (15–18)	17 (15–18)	−1
Malden	45,102	469	19 (17–22)	2,471	5	16 (15–17)	18 (16–20)	−1
Newton (462/466)	6,834	104	7 (6–8)	430	6	6 (4–8)	6 (4–8)	−1
Allston/Brighton	60,028	1,363	12 (11–14)	1,968	3	11 (9–12)	11 (9–14)	−1
Mattapan	19,623	495	14 (12–16)	1,335	7	12 (10–14)	13 (9–17)	0
Central Boston	19,332	677	9 (8–10)	2,547	13	9 (8–10)	8 (7–10)	0
Newton (459/467)	23,042	536	6 (5–7)	3,274	14	5 (4–6)	6 (5–7)	0
Newton (460/465)	15,927	289	7 (6–8)	1,281	8	6 (5–8)	8 (5–10)	1
Newton (458)	9,783	148	7 (6–9)	1,019	10	8 (6–9)	8 (6–11)	1
Newton (461/464/468)	11,299	223	5 (4–6)	1,036	9	7 (5–8)	7 (5–9)	2
Roslindale	24,474	905	13 (11–14)	3,375	14	14 (13–15)	15 (14–17)	3
Hyde Park	20,911	774	15 (13–17)	3,182	15	15 (14–16)	18 (16–20)	3
Jamaica Plain	38,146	1,402	11 (10–13)	5,665	15	14 (13–15)	15 (13–16)	4
Charlestown	12,371	543	12 (11–14)	4,015	32	19 (17–20)	18 (16–20)	6
Total or average	877,943	20,475	13	77,529	10	11	11	−2

Abbreviation: CI, confidence interval.

a Numbers following community names represent zip codes, which have a leading “02” omitted.

b Adult population estimates derived from the 2000 US Census.

c Population-based estimates derived from the Behavioral Risk Factor Surveillance System.

d Market share was calculated by dividing the number of Practice-Based Research Network patients by the census population aged 18 years or older in that neighborhood.

e Crude smoking prevalence was calculated by dividing the number of smokers by the total number of patients in each neighborhood.

f Standardized smoking prevalence was calculated by standardizing the age, sex, and race/ethnicity of Practice Based Research Network patients according to the neighborhood makeup according to the 2000 Census.

Community Name	Zip Code	Rate, %
East Boston	02128	17.4
Charlestown	02129	12.2
South Boston	02210	21.3
South Boston	02127	21.3
Central	02109	8.7
Central	02110	8.7
Central	02111	8.7
Central	02114	8.7
Back Bay/Beacon Hill	02108	12.1
Back Bay/Beacon Hill	02199	12.1
South End	02116	15.0
South End	02118	15.0
Fenway-Kenmore	02115	11.9
Fenway-Kenmore	02215	11.9
Allston/Brighton	02134	12.2
Allston/Brighton	02135	12.2
Allston/Brighton	02136	12.2
Allston/Brighton	02146	12.2
Jamaica Plain	02120	11.1
Jamaica Plain	02130	11.1
Roxbury	02119	17.4
Roxbury	02121	17.4
North Dorchester	02125	18.2
South Dorchester	02122	15.6
South Dorchester	02124	15.6
Mattapan	02126	13.6
Roslindale	02131	12.7
West Roxbury	02132	14.5
Hyde Park	02136	14.8
Cambridge	02138	7.3
Cambridge	02139	10.5
Cambridge	02142	10.5
Cambridge	02140	9.1
Cambridge	02141	15.2
Newton	02458	7.1
Newton	02460	6.9
Newton	02465	6.9
Newton	02459	5.6
Newton	02467	5.6
Newton	02462	6.7
Newton	02466	6.7
Newton	02461	5.1
Newton	02464	5.1
Newton	02468	5.1
Quincy	02169	18.5
Quincy	02170	13.5
Quincy	02171	13.5
Brookline	02445	8.3
Brookline	02446	8.3
Malden	02148	18.9
Revere	02151	25.9
Chelsea	02150	20.8
Needham	02492	7.5
Needham	02493	7.5
Needham	02494	7.5

**Figure 2:**
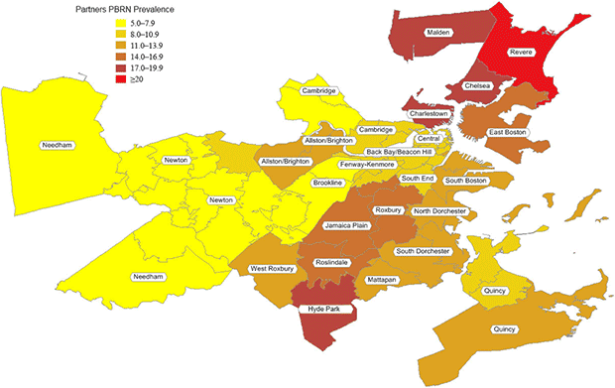
Practice-Based Research Network smoking prevalence in 32 Boston Neighborhoods. **Table:** 

**Figure 3:**
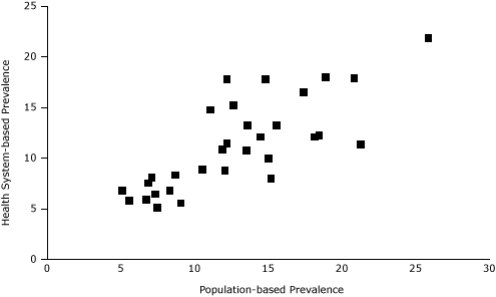
Scatter plot of Practice-Based Research Network (PBRN) smoking prevalence and population-based neighborhood prevalence of smoking for 32 Boston neighborhoods (%). **Table:** 

According to standardized PBRN data, the 32 neighborhoods had an overall smoking prevalence of 11%, ranging from 5% to 22% (Table 2, Figure 2). Compared with BRFSS estimates, the standardized PBRN estimates averaged 2 percentage points lower (standard deviation, 3 percentage points), and the differences ranged from −10 percentage points (South Boston; 11% PBRN prevalence and 21% BRFSS prevalence; 7% PBRN market share) to +6 percentage points (Charlestown; 18% PBRN prevalence and 12% BRFSS prevalence; 32% PBRN market share with a neighborhood Community Health Center). The PBRN estimates were lower by more than 5 percentage points in 5 neighborhoods (Table 2, Figure 3).

Community Name	Zip Code	Rate, %
East Boston	02128	16.4
Charlestown	02129	17.8
South Boston	02210	11.3
South Boston	02127	11.3
Central	02109	8.3
Central	02110	8.3
Central	02111	8.3
Central	02114	8.3
Back Bay/Beacon Hill	02108	8.7
Back Bay/Beacon Hill	02199	8.7
South End	02116	10
South End	02118	10
Fenway-Kenmore	02115	10.9
Fenway-Kenmore	02215	10.9
Allston/Brighton	02134	11.4
Allston/Brighton	02135	11.4
Allston/Brighton	02136	11.4
Allston/Brighton	02146	11.4
Jamaica Plain	02120	14.7
Jamaica Plain	02130	14.7
Roxbury	02119	16.5
Roxbury	02121	16.5
North Dorchester	02125	12.1
South Dorchester	02122	13.2
South Dorchester	02124	13.2
Mattapan	02126	13.2
Roslindale	02131	15.2
West Roxbury	02132	12.1
Hyde Park	02136	17.7
Cambridge	02138	6.4
Cambridge	02139	8.9
Cambridge	02142	8.9
Cambridge	02140	5.5
Cambridge	02141	8
Newton	02458	8.1
Newton	02460	7.5
Newton	02465	7.5
Newton	02459	5.7
Newton	02467	5.7
Newton	02462	5.9
Newton	02466	5.9
Newton	02461	6.8
Newton	02464	6.8
Newton	02468	6.8
Quincy	02169	12.2
Quincy	02170	10.7
Quincy	02171	10.7
Brookline	02445	6.7
Brookline	02446	6.7
Malden	02148	18
Revere	02151	21.9
Chelsea	02150	17.9
Needham	02492	5.1
Needham	02493	5.1
Needham	02494	5.1

Community Name	Zip Code	BRFSS Prevalence, %	Partners PBRN Prevalence, %
Newton	02461	5.1	6.8
Newton	02464	5.1	6.8
Newton	02468	5.1	6.8
Newton	02459	5.6	5.7
Newton	02467	5.6	5.7
Newton	02462	6.7	5.9
Newton	02466	6.7	5.9
Newton	02460	6.9	7.5
Newton	02465	6.9	7.5
Newton	02458	7.1	8.1
Cambridge	02138	7.3	6.4
Needham	02492	7.5	5.1
Needham	02493	7.5	5.1
Needham	02494	7.5	5.1
Brookline	02445	8.3	6.7
Brookline	02446	8.3	6.7
Central	02109	8.7	8.3
Central	02110	8.7	8.3
Central	02111	8.7	8.3
Central	02114	8.7	8.3
Cambridge	02140	9.1	5.5
Cambridge	02139	10.5	8.9
Cambridge	02142	10.5	8.9
Jamaica Plain	02120	11.1	14.7
Jamaica Plain	02130	11.1	14.7
Fenway-Kenmore	02115	11.9	10.9
Fenway-Kenmore	02215	11.9	10.9
Back Bay/Beacon Hill	02108	12.1	8.7
Back Bay/Beacon Hill	02199	12.1	8.7
Charlestown	02129	12.2	17.8
Allston/Brighton	02134	12.2	11.4
Allston/Brighton	02135	12.2	11.4
Allston/Brighton	02136	12.2	11.4
Allston/Brighton	02146	12.2	11.4
Roslindale	02131	12.7	15.2
Quincy	02170	13.5	10.7
Quincy	02171	13.5	10.7
Mattapan	02126	13.6	13.2
West Roxbury	02132	14.5	12.1
Hyde Park	02136	14.8	17.7
South End	02116	15.0	10
South End	02118	15.0	10
Cambridge	02141	15.2	8
South Dorchester	02122	15.6	13.2
South Dorchester	02124	15.6	13.2
East Boston	02128	17.4	16.4
Roxbury	02119	17.4	16.5
Roxbury	02121	17.4	16.5
North Dorchester	02125	18.2	12.1
Quincy	02169	18.5	12.2
Malden	02148	18.9	18
Chelsea	02150	20.8	17.9
South Boston	02210	21.3	11.3
South Boston	02127	21.3	11.3
Revere	02151	25.9	21.9

## Discussion

Neighborhood-level smoking prevalence can be estimated using EHR data from a PBRN, collected in the routine course of clinical care. Generally, PBRN estimates were slightly lower than the BRFSS estimates, but they were higher in some neighborhoods in which the PBRN had higher market penetration.

PBRN smoking prevalence estimates may have been generally lower for several reasons. Practices did not have smoking status documented for 100% of patients. Though not directly comparable, our 73% documentation rate compares favorably to the BRFSS response rate of 48%, a rate that is declining (10). In addition, clinicians preferentially document the smoking status of smokers. Efforts to increase smoking status documentation, including local and national incentives and EHR reminders, preferentially add nonsmokers, leading to small changes in the measured smoking prevalence. Our previous intervention to increase smoking status documentation served to increase the rate of documentation only for patients who were former smokers and never smokers; there was no significant change in the proportion of documented smokers (11). The discrepancies between PBRN and BRFSS estimates may also be attributable to the differential coverage of the populations by PBRN and BRFSS. Also, our PBRN patient population differs from the greater Boston area adult population, the sampling frame for the BRFSS, by having a lower percentage of Medicaid beneficiaries, more women than men, and likely healthier or more health-conscious patients (12).

Other studies have noted similarities and differences between health system–based and population-based estimates of smoking prevalence. A study in Leicester, England, found that General Practice notes, computerized and manual, tended to overestimate population smoking prevalence (13). On a larger scale in the UK, a comparison of an EHR-based PBRN, containing approximately 6% of the UK population, to the population-based General Lifestyle Survey showed excellent agreement both nationally and regionally (14,15). However, this analysis is not geographically granular enough to allow targeting of neighborhood resources. In the United States, commercial EHRs with broad national penetration may have greater potential to make regional and national estimates of acute and chronic conditions, as well as behavioral risk factors (16).

Using PBRN or health system data to measure the neighborhood prevalence of smoking offers several advantages over traditional, population-based methods. First, the data are already collected as part of routine clinical care, so their collection is less expensive than using a separate infrastructure to conduct population-based surveys. Second, for small-area estimation, as our data show, the sample sizes available in PBRN data are much larger, resulting in smaller standard errors (although relative to neighborhood population, even the PBRN estimates might be considered small), which may allow for the targeting of community-based interventions in smaller areas than is feasible using the BRFSS. Third, given the larger sample size and collection during routine clinical care, the data are potentially more current than population-based surveys, for which time is needed for data to become available (14). Fourth, because data were taken from an EHR, the potential exists for smoking status to be linked to a richer data set consisting of other behaviors, comorbidities, medications, and health outcomes for varied patients (17). Similarly, EHR-based measurements can be linked with practice-based treatment interventions (11,18–20). Finally, the emergence and implementation of EHR data standards as part of the national Meaningful Use EHR Incentive program will allow for information pooling across multiple health systems and encourage routine documentation of smoking status as it did for the General Practice pay-for-performance contract in Britain (21). Some of the inherent data problems associated with market share of this single PBRN could be resolved by pooling data from multiple health systems.

Despite these advantages to PBRN and health system data, advantages exist to population-based estimates, such as those derived from BRFSS. Obviously, despite low and declining response rates, the BRFSS is population-based and can provide estimates regardless of whether an individual seeks care through a particular health system or seeks health care at all. The BRFSS is not dependent on “market share” to get more accurate estimates, is not dependent on the presence of health care facilities in neighborhoods, and is not subject to health system peculiarities that may limit the generalizability of PBRN data. Second, although it was not intended to provide the small-scale prevalence we calculated, the BRFSS is consistently administered across the United States. Third, the BRFSS potentially provides greater consistency over time. Although standards are emerging for the structure of EHR data, the BRFSS data are collected with greater attention to consistency in definitions and measurement. Population-based surveys are not free from bias, however. For example, smokers generally have lower response rates to surveys than do nonsmokers (13). Finally, patients may be more likely to report negative health behaviors like smoking to health care providers than via a population survey.

Our analysis suggests that PBRNs and health system data can be used to guide community resources to neighborhoods with greater need. However, an understanding of the limitations of estimates derived from nonpopulation-based sources, for example, data from a single health system with low market penetration, cannot be used for small-area estimation, because the results are potentially biased because of insufficient population coverage and selection bias.

The true public health benefit of using health system–based risk factor information will come from combining data from multiple health systems, which requires a change in view of health system data as a public health resource. A convergence of health system data with population-based results should be seen more often as documentation improves (14). Health system data could be part of a systematic surveillance system that would help in understanding the effectiveness of tobacco control programs (22), particularly with mandated reporting of behavioral risk factors such as tobacco use, obesity, alcohol use, and others (23). Such a system could afford greater opportunity for collaboration and synergy between health care systems, public health departments, community organizations, and other community-based resources. In effect, this may encourage the surrounding community to take a greater stake in the health system and the health system to take a greater stake in the surrounding community.
